# Geoffrey St. Hilaire

**Published:** 1844-08-10

**Authors:** 


### RECORD OF MEDICAL SCIENCE.

Dr. Hope, the distinguished Professor of Chemistry in the University of Edinburgh, died on the 13th of June. Dr. William Gregory, of the University of Aberdeen, has been appointed to the vacant chair.

### GEOFFREY ST. HILAIRE.

By the late European Journals we learn that this distinguished naturalist and philosopher is no more. His interment took place on the 22d ultimo, at the beautiful Cemetery of Pere La Chaise, Paris.

"The funeral, says the London Medical Times, was one of those imposing things that occur, in reference to scientific men, only in France. The chief mourner was the distinguished son. The pall was supported by Baron Dupin, President of the Academy of Science, Ferrus, the President of the Academy of Medicine, and Chevreul, the Director of the Museum of Natural History. An immense crowd, including among others the most eminent medical, scientific, and literary men of Paris, followed the hearse. The moment the coffin entered Pere La Chaise, a body of the populace, some of the ordinary labourers at the Jardin des Plants, took the horses from the hearse, and bore the corpse on their shoulders to the grave. The accompanying multitude, we are assured, were much touched by this proof of attachment on the part of the labourers; and its effect was not a little enhanced by the eloquent funeral addresses delivered by Dumas, Chevreul, Pariset, Serres, and Lakanel." M. St. Hilaire was the favourite pupil of the celebrated Abbe Hoüy, and had Daubertin for a patron, and Cuvier as his assistant and co-labourer. He was distinguished for his general science, but more particularly as a zoologist and general anatomist. For some years before his death he was blind. One daughter and a son, who has already attained to high distinction, remain as the heirs of his fortune and his fame.

